# Trauma in childhood is associated with greater pain catastrophizing but not anxiety sensitivity: a cross-sectional study

**DOI:** 10.1097/PR9.0000000000001083

**Published:** 2023-06-27

**Authors:** Ariane Delgado-Sanchez, Christopher Brown, Christiana Charalambous, Manoj Sivan, Anthony Jones

**Affiliations:** a Human Pain Research Group, Division of Human Communication, Development, and Hearing, University of Manchester, Manchester, United Kingdom; b Institute of Population Health, University of Liverpool, Liverpool, United Kingdom; c Department of Mathematics, The University of Manchester, Manchester, United Kingdom; d Leeds Institute of Rheumatic and Musculoskeletal Medicine, University of Leeds, Leeds, United Kingdom

**Keywords:** Vulnerability, Catastrophizing, Anxiety sensitivity, Trauma, Pain

## Abstract

**Introduction:**

Adverse life experiences have been identified as a possible vulnerability factor for chronic pain. This association could result from the effect of trauma on the psychological state of individuals. Previous studies found childhood trauma to be associated with pain catastrophizing and anxiety sensitivity, both of which have been associated with an increased risk of chronic pain. However, it is unknown whether trauma in adulthood affects these variables and whether the effect on pain catastrophizing is independent of confounds such as depression and anxiety.

**Objectives:**

To test the effect of childhood and adulthood trauma on pain catastrophizing and anxiety sensitivity whilst controlling for depression and anxiety.

**Methods:**

In the current study, we conducted an online survey in the United Kingdom in a chronic pain sample (N = 138; 123 women; age range 19–78). We analysed whether there is an association between different types of trauma (both in childhood and through the lifespan), pain catastrophizing, and anxiety sensitivity while controlling for anxiety and depression.

**Results:**

We found that childhood trauma (particularly emotional abuse) significantly predicts pain catastrophizing, even when controlling for depression and anxiety, whereas it did not have a significant effect on anxiety sensitivity. Trauma through the lifespan (not childhood) did not have a significant effect on anxiety sensitivity nor did it have a significant effect on pain catastrophizing.

**Conclusions:**

Our results show that the life stage in which trauma occurs is key in its psychological effects on patients with chronic pain. Furthermore, it shows that trauma affects some psychological variables but not others.

## Abstract 2

Supplemental Digital Content is Available in the Text.

Trauma during childhood but not adulthood affects pain catastrophizing. Emotional trauma is the most impactful in this association. Trauma has no impact on anxiety sensitivity.

## 1. Introduction

Chronic pain affects between 35% and 51.3% of the UK population.^7^ A better understanding of the factors leading to the development of chronic pain may inform better interventions for its treatment and prevention. Adverse life experiences have been identified as a possible vulnerability factor for chronic pain with several studies finding a higher rate of childhood trauma (in the form of abuse and neglect) in those suffering from pain disorders,^4^ as well as an association between lifetime exposure to psychological trauma and pain outcomes.^15^ One possible causal factor for this association is through the effect of trauma on the psychological state of patients. Trauma could influence the way individuals process information, particularly by increasing levels of pain catastrophizing (exaggerated negative emotions and cognitions associated with pain^12^) and anxiety sensitivity (fear of sensations related to arousal^22^). Although distinct constructs (as demonstrated by their definitions), both of these variables have been associated with an increased risk of developing chronic pain.^23,24^

Some preliminary evidence supports the link between trauma and increased levels of these variables. One study found an association between childhood trauma and pain catastrophizing in a chronic pain sample.^18^ However, this study did not control for the effect of depression and anxiety,^18^ which are highly correlated with pain catastrophizing^16^ and anxiety sensitivity,^9,11^ respectively, as well as being comorbid with chronic pain.^5,26^ Moreover, anxiety and depression are also strongly linked with the experience of traumatic events.^10,14,19^ In fact, another investigation of healthy adolescents found a link between childhood trauma and anxiety sensitivity, with a moderating effect of anxiety.^13^ Consequently, the correlations found between pain catastrophizing and trauma could be the result of their mutual correlation with depression and anxiety. This leaves some uncertainty about pain catastrophizing being an independent mechanism through which traumatic events influence the onset of chronic pain. Moreover, investigations on anxiety sensitivity and trauma have mostly focused on healthy youth, raising the question of whether the results are generalisable to an adult chronic pain population. Furthermore, although a link has been made between chronic pain and both trauma during childhood^4^ and through the entire lifespan,^15^ the few studies on the effect of trauma on the psychological state of patients explore only the effect of trauma during childhood. Therefore, it is difficult to assess whether the mechanism of trauma–psychological state–pain is applicable to trauma at any life stage or only at certain critical periods.

In the present investigation, we aimed to establish whether there is a specific association between trauma (in childhood and through the lifespan) and the psychological variables relevant to chronic pain development. We explored the effect of childhood and lifetime trauma on pain catastrophizing and anxiety sensitivity while controlling for depression and anxiety in a chronic pain sample. We hypothesized that we would see a significant effect of trauma (at all stages) on pain catastrophizing and anxiety sensitivity, independently of anxiety and depression.

## 2. Methods

### 2.1. Participants

One hundred forty-six patients with chronic pain took part in the study. Recruitment was performed through adverts on social media and patient support groups. Particularly, the social media groups used were chronic pain support groups/pages; some of the groups identified as general groups for chronic pain sufferers, others were condition-specific (fibromyalgia and arthritis). The survey was completed online through the platform Qualtrics. Eight participants were excluded because they did not complete all the key questionnaires used for the analysis performed in this study, leaving the total sample at 138 participants (123 women, 14 men, 1 nonbinary; mean age 47.69 [±14.03], age range 19–78). Note that 25 of the participants did not give a response when asked about their age; consequently, these participants were excluded from the calculation of the descriptive statistics regarding age and the cases were dropped for the analysis in which age was included as a covariate. A power analysis (power: 0.80, alpha: 0.05) showed that the sample was enough to detect relationships of moderate effects in a multiple regression analysis with 5 predictors. Participants self-identified as patients with chronic pain and were asked to provide a diagnosis. 63.04% of the patients reported a diagnosis of fibromyalgia, 18.11% a diagnosis of some type of arthritis, 7.97% a diagnosis of myalgic encephalomyelitis, 6.52% a diagnosis of low back pain, 43.47% reported various other diagnoses, and 9.42% did not specify the chronic pain condition they suffered. In supplementary materials 1 (available at http://links.lww.com/PR9/A198), a table with all the reported diagnosis can be seen. The percentages do not add to 100% because of the high comorbidity of the different chronic pain disorders. The study received ethical approval by the University of Manchester University Research Ethics Committee 2 (UREC 2), and all participants gave written informed consent.

### 2.2. Materials

The following questionnaires were included in the survey:

(1) *Short Version of the Childhood Trauma Questionnaire (CTQ-S)*^1^: This questionnaire is a 5-point Likert scale that presents 28 items (25 clinical items and 3 validity items) to retrospectively measure the occurrence of stressful events in childhood. It is divided in 5 subscales: emotional abuse, physical abuse, sexual abuse, emotional neglect, and physical neglect. It provides a general score and a score per subscale.
(2) *The Trauma History Screen*^3^: This 12 item questionnaire measures the occurrence of stressful events through the life of individuals (including childhood). People who answer the questionnaire must answer yes/no to whether they have lived through the event. If participants gave a positive answer, they were asked to rate the emotional impact the event had on them on a scale from 0 to 10. To avoid overlapping between the results of this questionnaire and the CTQ-S, the 2 items that explicitly ask about childhood events (items 4 and 6) were removed from the final analysis and the general score was calculated based on the remaining 10 items. We obtained 2 scores from this scale: number of experienced events and total emotional impact of experienced traumatic events.
(3) *Pain Catastrophizing Scale*^20^: This scale is a 13 item 5-point Likert scale built to measure the construct of pain catastrophizing. The questionnaire has 3 subscales: rumination, magnification, and helplessness. It provides a general catastrophizing score and a score per subscale.
(4) *Anxiety Sensitivity Index-3 (ASI-3)*^22^: This scale is a 5-point Likert scale that presents 18 items to measure an individual's predisposition to anxiety. The questionnaire has 3 subscales: physical, cognitive, and social anxiety sensitivity. It provides a general anxiety sensitivity score and a score per subscale.
(5) *The Hospital Anxiety and Depression Scale*^27^: This scale is a short 12-item scale with 4 choices build to measure depression and anxiety. The scale provides 2 scores, one for anxiety and one for depression.

### 2.3. Statistical analysis

Statistical analysis was performed in R-4.0.5. To test the effect of childhood trauma and full lifespan trauma on pain catastrophizing and anxiety sensitivity, 5 independent regression models were created for each dependent variable.

First, models with age and the independent variables (childhood trauma and full lifespan trauma) were created to test their effect in isolation. Afterward, the models were completed by adding depression and anxiety as covariates to test whether the effects held when controlling for these variables. Two following subsections can be found with a detailed description of models for each dependent variable: pain catastrophizing and anxiety sensitivity.

### 2.4. Assumption checks and transformation

To check the suitability of parametric tests, the assumptions of the models were tested.

In the models in which pain catastrophizing was used as an outcome, the assumptions were met.

In the anxiety sensitivity models, deviances from normality and homogeneity were observed. The deviances in homogeneity were fixed by taking the square of the outcome variable. After this, for the models with anxiety sensitivity as an outcome, the assumptions were met in every model bar one (the baseline model with only age as a predictor for anxiety sensitivity that violated the normality assumption). The full assumption check outcomes for the models with slight deviances can be seen in supplementary materials 2 (available at http://links.lww.com/PR9/A198). Based on the results the data were considered suitable for parametric tests.

### 2.5. Pain catastrophizing

Two sets of nested models were created to test the effect of trauma on pain catastrophizing. The first set included 3 models: the first model just with age as a predictor; the second model with age and childhood trauma as predictors; and the third model with age, childhood trauma, and impact of trauma history (adulthood trauma) as predictors. These models served to test the effect of trauma at different timepoints on pain catastrophizing without controlling for depression and anxiety. To test whether the inclusion of new factors improved the prediction, models were compared using a likelihood ratio test.

The second set of models included the covariates of depression and anxiety, as well as the independent variables (trauma variables), to test the effects of trauma when controlling for the possible confounding effects of depression and anxiety. Once again, to quantify whether the inclusion of new variables improved prediction (and therefore analyse whether the independent variables of trauma explained the variance of pain catastrophizing further than with the effect of depression and anxiety), these models were also compared using the likelihood ratio test. Particularly, 4 models were compared, the first model included only age as a predictor; the second model included age, depression, and anxiety as predictors; the third model included age, anxiety, depression, and childhood trauma as predictors; and the last model included age, anxiety, depression, childhood trauma, and impact of trauma history (adulthood trauma) as predictors.

### 2.6. Anxiety sensitivity

The above-described models were repeated with anxiety sensitivity as an outcome instead of pain catastrophizing.

### 2.7. Childhood trauma subscale effect

Because previous research has encountered that some subscales of childhood trauma have a greater impact on pain catastrophizing than others, we ran one final model in which we included each of the subscales (emotional abuse, physical abuse, sexual abuse, emotional neglect, and physical neglect) as independent predictors instead of a total score of childhood trauma. We also included as predictors the covariates of age, depression, and anxiety.

Childhood trauma was not a significant predictor of anxiety sensitivity; therefore, we did not run the subscale analysis with it as an outcome.

## 3. Results

### 3.1. Descriptive statistics

The mean and standard deviations of the different measures are presented in Table 1.

**Table 1:** Descriptive statistics.

	Mean	SD
Age	47.69	14.03
Depression	9.62	3.94
Anxiety	11.78	4.54
Childhood trauma	49.04	19.72
Emotional abuse	12.29	5.83
Physical abuse	7.86	3.99
Sexual abuse	8.20	5.53
Emotional neglect	12.75	6.02
Physical neglect	7.93	3.51
Impact of trauma history (adulthood trauma)	18.07	14.04

### 3.2. Pain catastrophizing

The results showed that childhood trauma was a significant predictor of pain catastrophizing, even when controlling for depression and anxiety, whereas trauma through the lifetime was not a significant predictor (Table 2).

**Table 2:** Models with pain catastrophizing as outcome: summary and model comparison.

	Model name	Linear regression outcome			Likelihood ratio test		Effect of predictors			VIF
		*R* ^2^	F	*P*	Log likelihood	*P*	Predictor	β	*P*	
Set of nested models 1 (without anxiety and depression)	MPC1	0.05	7.22	0.008	−443.90		Age	−0.22	0.008	
	MPC2	0.14	10.85	<0.001	−437.29	<0.001	Age	−0.26	0.001	1.01
							Childhood trauma	0.20	<0.001	1.01
	MPC3	0.15	7.76	<0.001	−436.52	0.21	Age	−0.27	<0.001	1.02
							Childhood trauma	0.18	0.001	1.07
							Impact of trauma history	0.09	0.22	1.05
Set of nested models 2 (with anxiety and depression)	MPC4	0.38	23.97	<0.001	−418.83		Age	−0.11	0.10	1.15
							Depression	1.29	<0.001	1.45
							Anxiety	0.77	0.004	1.63
	MPC5	0.40	20.21	<0.001	−415.89	0.01	Age	−0.14	0.04	1.19
							Depression	1.17	<0.001	1.49
							Anxiety	0.71	0.007	1.64
							Childhood trauma	0.11	0.01	1.09
	MPC6	0.40	16.05	<0.001	−415.85	0.77	Age	−0.15	0.03	1.21
							Depression	1.17	<0.001	1.49
							Anxiety	0.70	0.009	1.70
							Childhood trauma	0.11	0.02	1.13
							Impact of trauma history	0.01	0.78	1.10

MPC, outcome pain catastrophizing; MPC1, predictor: age; MPC2, predictors: age and childhood trauma; MPC3, predictors: age, childhood trauma, and impact of trauma history; MPC4, predictors: age and anxiety depression; MPC5, predictors: age, anxiety depression, and childhood trauma; MPC6, predictors: age, anxiety depression, childhood trauma, and impact of trauma history; VIF, variance inflation factor.

The model comparisons showed that in the models without anxiety and depression, adding childhood trauma improved the fit of the model but adding impact of trauma history (adulthood trauma) did not. In the case of models with covariates, it showed that adding anxiety, depression, and childhood trauma improved the prediction but adding impact of trauma history (adulthood trauma) did not. In other words, childhood trauma was shown to be a significant predictor that explains a part of the pain catastrophizing variance independent to that explained by anxiety and depression; however, impact of trauma history (adulthood trauma) was not. A summary of the results is presented in Table 2. These results were corroborated by a joint regression model in which the correlation between pain catastrophizing and anxiety sensitivity was accounted for (supplementary materials 3, available at http://links.lww.com/PR9/A198).

### 3.3. Anxiety sensitivity

Results showed that childhood trauma (but not adulthood trauma) was a significant predictor of anxiety sensitivity, but this effect became nonsignificant when adulthood trauma was also considered, as well as when controlling for anxiety and depression. Interestingly, if the covariate of age was not included in the model, adulthood trauma was a significant predictor while childhood trauma was not. This effect also became nonsignificant when controlling for anxiety and depression (the analysis without age as a covariate can be found in the supplementary materials 4; furthermore, an analysis with only lifetime trauma impact as a predictor is included in supplementary materials 5, available at http://links.lww.com/PR9/A198). From the covariates, anxiety was a significant predictor but depression was not. A summary of the results can be found in Table 3.

**Table 3:** Models with anxiety sensitivity as outcome: summary and model comparison.

	Model name	Linear regression outcome			Likelihood ratio test		Effect of predictors			VIF
		*R* ^2^	F	*P*	Log likelihood	*P*	Predictor	β	*P*	
Set of nested models 1 (without anxiety and depression)	MAS1	0.06	8.66	0.003	−235.07		Age	−0.03	0.003	
	MAS2	0.09	7.04	0.001	−232.51	0.02	Age	−0.04	0.001	1.01
							Childhood trauma	0.02	0.02	1.01
	MAS3	0.10	5.49	0.001	−231.37	0.12	Age	−0.04	<0.001	1.02
							Childhood trauma	0.01	0.06	1.07
							Impact of trauma history	0.01	0.13	1.05
Set of nested models 2 (with anxiety and depression)	MAS4	0.48	36.67	<0.001	−199.89	<0.001	Age	0	0.57	1.15
							Depression	0.02	0.51	1.45
							Anxiety	0.29	<0.001	1.63
	MAS5	0.48	27.67	<0.001	−199.46	0.35	Age	0	0.47	1.19
							Depression	0.02	0.62	1.49
							Anxiety	0.29	<0.001	1.64
							Childhood trauma	0	0.36	1.09
	MAS6	0.48	21.93	<0.001	−199.46	0.95	Age	0	0.47	1.21
							Depression	0.02	0.62	1.49
							Anxiety	0.29	<0.001	1.70
							Childhood trauma	0	0.37	1.13
							Impact of trauma history	0	0.95	1.10

MAS, outcome anxiety sensitivity; MAS1, predictor: age; MAS2, predictors: age and childhood trauma; MAS3, predictors: age, childhood trauma, and impact of trauma history; MAS4, predictors: age and anxiety depression; MAS5, predictors: age, anxiety depression, and childhood trauma; MAS6, predictors: age, anxiety depression, childhood trauma, and impact of trauma history; VIF, variance inflation factor.

The model comparison showed that in the models without covariates, adding childhood trauma improved the model. In the case of models with covariates, it showed that neither childhood trauma nor impact of trauma history (adulthood trauma) improved the prediction. In other words, when controlling for anxiety and depression, trauma does not explain a significant part of the anxiety sensitivity variance. A summary of the results is presented in Table 3. These results were corroborated by a joint regression model in which the correlation between pain catastrophizing and anxiety sensitivity was accounted for (supplementary materials 3, available at http://links.lww.com/PR9/A198).

### 3.4. Childhood trauma subscale effect

Results showed that while controlling for depression, anxiety, and age, only 2 types of trauma significantly predicted the level of pain catastrophizing: emotional abuse and physical abuse (Table 4).

**Table 4:** Subscale analysis: effect of types of childhood trauma on pain catastrophizing.

*R* ^*2*^	F		*P*
0.46	13.16		<0.001
Predictors	**β**	***P***	**VIF**
Emotional abuse	0.61	0.04	3.79
Physical abuse	0.92	0.004	2.17
Sexual abuse	−0.04	0.77	1.28
Emotional neglect	−0.48	0.09	4.03
Physical neglect	−0.31	0.47	3.24
Depression	1.20	<0.001	1.50
Anxiety	0.77	0.002	1.67
Age	−0.11	0.10	1.27

VIF, variance inflation factor.

## 4. Discussion

In this study, we found that childhood trauma, but not trauma through the lifespan, significantly predicts the levels of pain catastrophizing in patients with chronic pain. Importantly, this work showed that this is true even when controlling for depression and anxiety. Furthermore, this is to the best of our knowledge the first account that shows that the period in which trauma occurs is a key in its effects on psychological variables important for chronic pain vulnerability. We found that childhood emotional abuse and physical abuse are the types of trauma that influence the levels of pain catastrophizing. Interestingly, sexual abuse, physical neglect, and emotional neglect did not show a significant effect. When it comes to anxiety sensitivity, we found that childhood trauma only had a significant effect on anxiety sensitivity when we did not control for the covariates of anxiety and depression, showcasing the importance of including these covariates in the study of the relationship between trauma and other psychological variables. Finally, the emotional impact of trauma through the lifespan (not childhood) did not have a significant effect on this variable.

The findings in our study support the previous preliminary evidence pointing to a link between childhood trauma and pain catastrophizing.^18^ The fact that the effect of childhood trauma is still significant when corrected for anxiety and depression suggests that this effect is not mediated by these states. Consequently, altering the psychological state of individuals through pian catastrophizing may be a possible mechanism through which childhood trauma increases chronic pain vulnerability.

It is particularly interesting that only 2 types of childhood trauma (emotional abuse and physical abuse) had an effect on pain catastrophizing, whereas sexual abuse, physical neglect, and emotional neglect showed no significant effect. This, at first, may seem counterintuitive because some would classify sexual abuse and physical neglect as the more severe types of abuse. Nevertheless, it is consistent with recent findings in the literature. In the study by Sansone et al. (2013), they found that when running a multivariate analysis with all the variables (instead of multiple univariate analyses) only emotional abuse was a significant predictor of pain catastrophizing. Several studies on the effect of the different types of abuse on the psychological state of individuals have found that emotional abuse has a bigger impact than other forms of abuse.^6,8^ Perhaps this higher impact of emotional abuse could be due to its greater effect on the development of emotional regulation.^2^ Another possible explanation could be the higher chronification of this type of abuse as a consequence of it going undetected or even the normalization by others or even the victim (due to gaslighting or victim blaming), as well as due to the more subtle nature of the abuse that hampers the management of psychological consequences.

There are a number of potential public health implications of these findings. Emotional abuse is a type of abuse that often goes undetected or unchallenged, which could lead to a higher proportion of the population being at risk. If confirmed by larger prospective studies, emotional abuse should be considered as an important risk factor for pain catastrophizing. Because pain catastrophizing is correlated with chronification of pain and disability,^21^ strategies for prevention and treatment of chronic pain may need to take emotional abuse history into account.

However, our findings (and similar findings from other studies) should not be interpreted as a complete lack of effect of other types of childhood abuse. Our results show that physical abuse also has an effect on pain catastrophizing and therefore may contribute to the risk for developing chronic pain. It should be emphasised that the absence of significant effects of sexual abuse, physical neglect, and emotional neglect on pain catastrophizing does not exclude their effect on developing chronic pain through alternative mechanisms such as allostatic load^17^ or by the effect on other psychological and cognitive factors such as negative bias or somatic awareness.

The lack of effect of childhood trauma on anxiety sensitivity is an interesting finding that contradicts some previous research.^13^ Anxiety sensitivity has been at the centre of many chronic pain vulnerability models, but there has been discussion about its intersubject variability. Some authors have proposed that both genetic and environmental factors could have an effect, with genetic variables presenting a long-term effect and environmental factors being responsible for short-term variability.^25^ Perhaps this would explain why Martin et al. (2014) found an effect of childhood trauma while we did not because their study was conducted on adolescents and ours on adults with a mean age of 47.67 [±13.90]. This would also explain our finding of the small effect of childhood abuse when we did not control for the covariates of depression and anxiety, as well as the small effect of lifespan trauma in the models that did not control for age. The time since trauma occurred could be having an impact on the results, and therefore, future research could explore this by recording the time elapsed between the traumatic event and the questionnaire or through longitudinal studies.

Our findings suggest that the time in which people are exposed to trauma is key to assess whether this will have an effect on the levels of pain catastrophizing, childhood being a critical period for trauma to influence the psychological state of individuals. Several factors could explain this time sensitivity such as the lower ability of children to understand the occurrences, or the fact that during childhood, individuals are still developing their personality and biology, making changes through this period more likely to affect health outcomes in the long term.

The lack of effect of trauma during adulthood on pain catastrophizing and anxiety sensitivity does not exclude an effect on pain. Some chronic pain vulnerability models propose that the development of chronic pain is a combination of diathesis (vulnerability) and the occurrence of a stressor.^23^ Our findings are an indication of trauma during adulthood not being a clear influence on diathesis (at least through pain catastrophizing and anxiety sensitivity); nevertheless, it could still be acting as a stressor that can trigger the development of pain.

There were some limitations of our design. This was a cross-sectional study with a mixed sample of patients with chronic pain. Future research should try to corroborate our findings with a longitudinal design and by dividing the sample based on the diagnosis and possibly extent of pain experienced by the patients. Furthermore, participants were warned about the questions regarding trauma before participation (in the study advert), which may have mean that those highly affected by trauma decided to not take part potentially skewing the population we sampled from. Future research should try to overcome these limitations perhaps by recruiting the sample through pain clinics or clinicians who participants may already feel comfortable discussing these issues with. Finally, larger prospective longitudinal studies would be useful to further study the temporal dynamics of the interactions between trauma and anxiety sensitivity, as well as the effect of emotional abuse on chronic pain and how the effects of the former on the latter might be better prevented or treated.

In summary, our findings show that one of the pathways through which trauma could be affecting chronic pain is by increasing the levels of pain catastrophizing but not anxiety sensitivity. Furthermore, this effect is only apparent for trauma that occurs during the critical period of childhood and only through certain types of trauma, particularly emotional abuse. This shows that psychological interventions in patients with chronic pain may need to pay special attention to the effects of emotional abuse during childhood, which may be different to the ones caused by other types of trauma.

## Disclosures

The authors have no conflict of interest to declare.

## Appendix A. Supplemental digital content

Supplemental digital content associated with this article can be found online at http://links.lww.com/PR9/A198.
