# Supplementary material for: Trauma in childhood is associated with greater pain catastrophizing but not anxiety sensitivity: a cross-sectional study
Source: Pain Rep. 2023 Jun 27;8(4):e1083. doi: 10.1097/PR9.0000000000001083 (PMC10306427; doi:10.1097/PR9.0000000000001083)
Supplement: Supplementary file 1 [file painreports-8-e1083-s001.pdf]

Supplementary materials 1: Diagnoses reported by patients

Supplementary table 1: Diagnoses reported by patients

Diagnosis	Number of patients with diagnosis	Percentage of sample (138)
Fibromyalgia	87	63.04%
Osteoarthritis	13	9.42%
Arthritis (type unspecified)	7	5.07%
Psoriatic Arthritis	3	2.17%
Inflammatory Arthritis	1	0.72%
Rheumatoid Arthritis	1	0.72%
Diagnosis non specified	13	9.42%
Myalgic encephalomyelitis/Chronic Fatigue	11	7.97%
Low Back Pain	9	6.52%
Failed Back Surgery Syndrome	5	3.62%
Migraine	4	2.89%
Hypermobility	4	2.89%
Thoracic Outlet Syndrome	3	2.17%
Sciatica	3	2.17%
Ehlers–Danlos syndrome	2	1.44%
Plantar Facitis	2	1.44%
Chronic Cluster Headaches	2	1.44%
Endometriosis	2	1.44%
Scoliosis	2	1.44%
Complex Regional Pain Syndrome	2	1.44%
Degenerative Disc Disease	2	1.44%
Sjögren's syndrome	2	1.44%
Prolapsed Spinal Disc	2	1.44%
Costocondritis	2	1.44%
Stenosis	1	0.72%
Ankylosing Spondylitis	1	0.72%
Adhesive Arachnoiditis	1	0.72%
Hashimotos	1	0.72%
Brachial Neuritis	1	0.72%
Sacroiliitis	1	0.72%
Spinal Injury	1	0.72%
Atypical Facial Pain	1	0.72%
Adenomyosis	1	0.72%
Post-Thrombotic Syndrome	1	0.72%
Chronic Neuropathic Pain	1	0.72%
Post hip replacement Chronic Pain	1	0.72%

Functional Neurological Disorder	1	0.72%
Tendonitis	1	0.72%
Bursitis	1	0.72%
Trapped Nerve in Shoulder	1	0.72%
Back pain (location unspecified)	1	0.72%
Chronic Nerve Hypersensitivity	1	0.72%
Post Herpetic Neuralgia	1	0.72%
Trigeminal Neuralgia	1	0.72%
Myofascial Pain Syndrome	1	0.72%

Supplementary materials 2:

From the models tested the following showed potential violations of some assumption:

-Anxiety Sensitivity ~ Age

The assumption diagnostic for the model can be seen here.

Anxiety Sensitivity ~ Age

Normality:

- Shapiro Wilk (0.9734; p=0.0234)

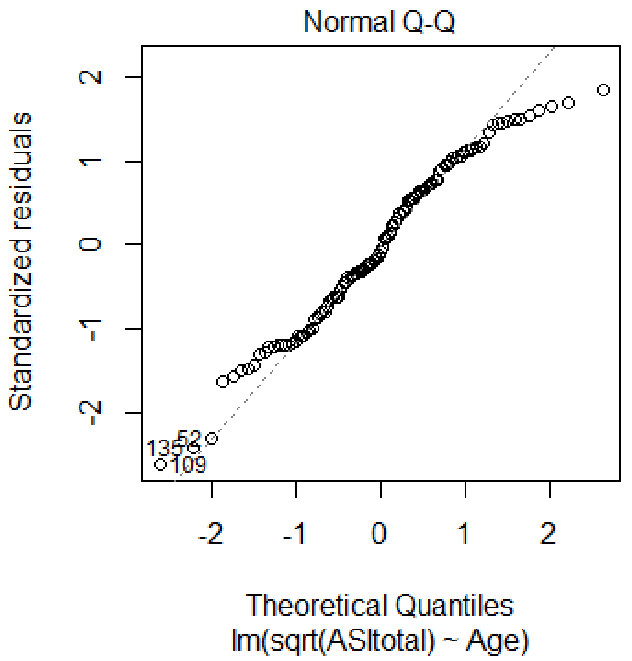

Figure 9: Q-Q plot

Multicolinearity:

- NA: Only one variable

Independence:

- Durbin Watson: 2.11

Linearity:

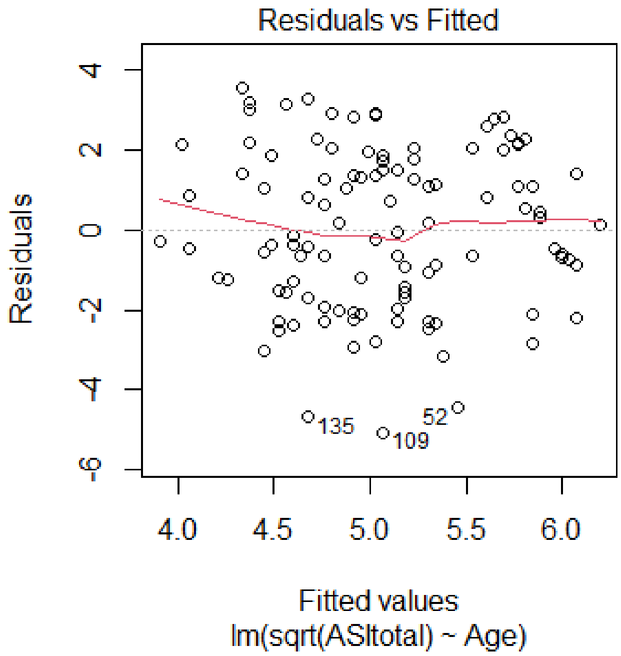

Figure 10: Residuals vs Fitted graph

Homoscedasticity:

- Breusch-Pagan: 0.98; p=0.32

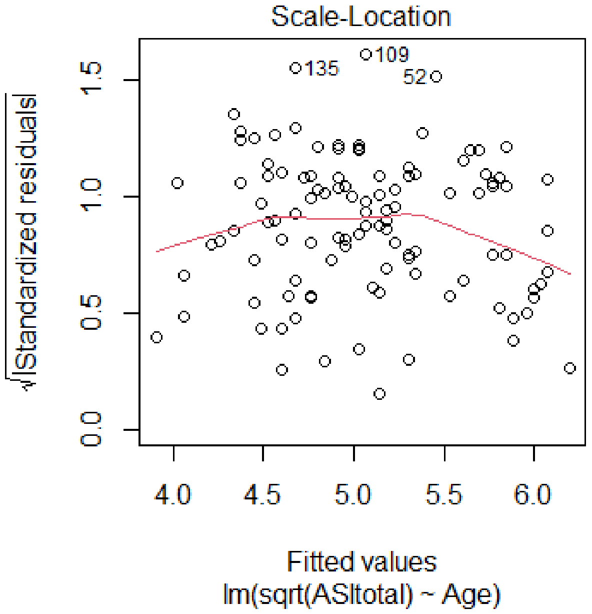

Figure 11: Fitted values vs Standardized residuals

Cook's Distance

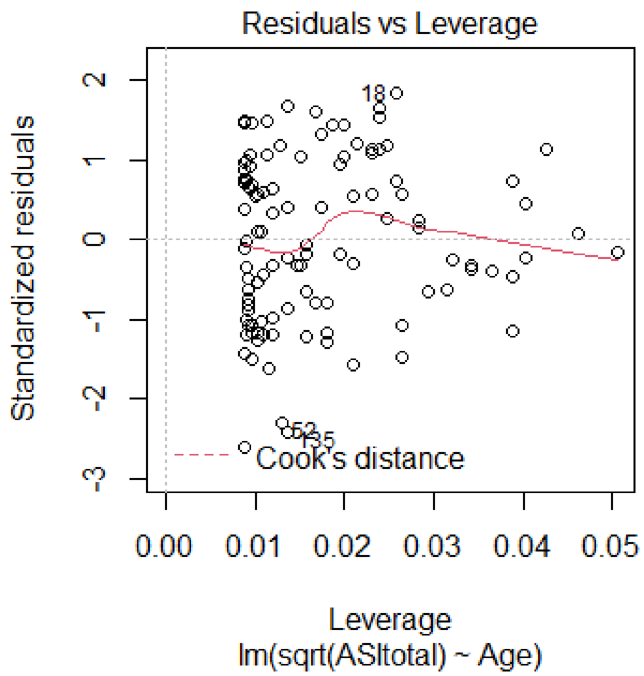

Figure 12: Residuals vs Leverage

Supplementary materials 3: Joint regression results

A joint regression in which the two dependent variables were included was carried out through the GJTM package in R.

Once again, five different models were calculated.

- Childhood trauma without control variables model (pain catastrophizing ~ age + childhood trauma, anxiety sensitivity ~ age + childhood trauma): This was a joint regression model with childhood trauma as a predictor and both pain catastrophizing and anxiety sensitivity as outcomes.
- Full lifespan trauma without control variables model (pain catastrophizing ~ age + childhood trauma + impact of trauma history, anxiety sensitivity ~ age + childhood trauma + impact of trauma history): This model was a joint multiple regression model with childhood trauma and trauma history impact as predictors and both pain catastrophizing and anxiety sensitivity as outcomes.
- Covariate only model (pain catastrophizing ~ age + depression + anxiety, anxiety sensitivity ~ age + anxiety + depression): This model was a joint multiple regression model with the control variables as predictors and both pain catastrophizing and anxiety sensitivity as outcomes.
- Childhood trauma model (pain catastrophizing ~ age + depression + anxiety + childhood trauma, anxiety sensitivity ~ age + anxiety + depression + childhood trauma): This model was a joint multiple regression model with depression,

anxiety and childhood trauma as predictors and both pain catastrophizing and anxiety sensitivity as outcomes.

- Full lifespan trauma model (pain catastrophizing ~ age + depression + anxiety + childhood trauma + impact of trauma history, anxiety sensitivity ~ age + anxiety + depression + childhood trauma + impact of trauma history): This model was a joint multiple regression model with depression, anxiety, childhood trauma and trauma history impact as predictors, and both pain catastrophizing and anxiety sensitivity as outcomes.

In table 2 the BIC (Bayesian Information Criterion) and AIC (Akaike information criterion) associated with each model can be seen. These criteria measure the fit of the model and their ability to predict future data, as well as, penalising the models with higher complexity. A lower value is an indicator of a better model fit.

Results show the same pattern as the one observed with the two independent regressions. In the models without anxiety and depression as covariates, the model was improved by adding childhood trauma but nor by adding impact of trauma history. In the case of models with covariates, the AIC indicated that the model was improved when adding childhood trauma but not when adding the variable impact of trauma history. The BIC did not show such convincing results of the overall benefits of including childhood trauma (supplementary table 2). This could be due to the lack of effect of childhood trauma on anxiety sensitivity. When looking at the effect of each predictor on the different outcome variables this is corroborated. The same way as with the independent models childhood trauma was shown to be a significant predictor of pain catastrophizing even when controlling for depression and anxiety, but impact of lifetime trauma was not. Meanwhile, childhood trauma showed a significant effect on anxiety sensitivity only until we controlled for the effect of lifetime trauma. Furthermore, in the models controlling for anxiety and depression childhood trauma did not show a significant effect (supplementary table 3).

Supplementary table 2: Fit of joint regression models

**Set of nested models 1 (without anxiety and depression)**			**Set of nested models 2 (with anxiety and depression)**		
**Model**	**BIC**	***AIC***	**Model**	**BIC**	***AIC***
JM1	1859.871	1840.78	JM4	1789.822	1759.82
JM2	1856.084	1831.538	JM5	1793.378	1757.922
JM3	1862.634	1832.633	JM6	1802.713	1761.802

Note:; JM= Joint regression model with pain catastrophizing and anxiety sensitivity as outcomes; JM1= predictors: age; JM2= predictors: age, childhood trauma; JM3= predictors: age, childhood trauma, impact of trauma history; JM4=predictors: age, anxiety depression; JM5= predictors: age, anxiety depression, childhood trauma; JM6= predictors: age, anxiety depression, childhood trauma, impact of trauma history.

Supplementary table 3: Predictor significance for pain catastrophizing and anxiety sensitivity in joint models

	Model name	Predictors	*Outcome: Pain catastrophizing*		*Outcome: Anxiety sensitivity*	
			**β**	***p***	**β**	***p***
**Set of nested models 1 (without anxiety and depression)**	JM1	Age	-0.22	.006	-0.38	0.003
	JM2	Age	-0.26	< .001	-0.42	< .001
		Childhood trauma	0.20	< .001	0.19	0.02
	JM3	Age	-0.27	< .001	-0.43	< .001
		Childhood trauma	0.18	< .001	0.16	0.06
		Impact of Trauma History	0.09	.21	0.21	0.09
**Set of nested models 2 (with anxiety and depression)**	JM4	Age	-0.11	0.09	-0.06	0.52
		Depression	1.29	< .001	0.29	0.47
		Anxiety	0.77	.003	2.86	< .001
	JM5	Age	-0.14	0.03	-0.08	0.43
		Depression	1.17	< .001	0.23	0.58
		Anxiety	0.71	.005	2.83	< .001
		Childhood trauma	0.11	0.01	0.06	0.36
	JM6	Age	-0.15	0.03	-0.08	0.41
		Depression	1.17	< .001	0.23	0.57
		Anxiety	0.70	.007	2.81	< .001
		Childhood trauma	0.11	0.01	0.05	0.39
		Impact of Trauma History	0.01	0.77	0.02	0.76

Note:; JM= Joint regression model with pain catastrophizing and anxiety sensitivity as outcomes; JM1= predictors: age; JM2= predictors: age, childhood trauma; JM3= predictors: age, childhood trauma, impact of trauma history; JM4=predictors: age, anxiety depression; JM5= predictors: age, anxiety depression, childhood trauma; JM6= predictors: age, anxiety depression, childhood trauma, impact of trauma history.

Supplementary materials 4: Effect of predictors on Anxiety Sensitivity without controlling for the covariate of age

Anxiety sensitivity

When age was not included in the model as a covariate, results showed that lifetime trauma (but not childhood trauma) was a significant predictor of anxiety sensitivity, but, this effect disappeared when controlling for anxiety. From the covariates, anxiety was a significant predictor but depression was not. A summary of the results can be found in supplementary table 4.

Supplementary table 4: Models with anxiety sensitivity as outcome and without age as a covariate (summary and model comparison)

	Model name	Linear Regression outcome			Likelihood ratio test		Effect of predictors		
		**R^2**	**F**	**p**	**Log likelihood**	**p**	**Predictor**	**β**	**p**
**Set of nested models 1 (without anxiety and depression)**	NoAge MAS2	.01	2.859	.093	-289.53		Childhood trauma	0.01	.093
	NoAge MAS3	.03	3.581	.030	-287.40	.038	Childhood trauma	0.01	.256
							Impact of Trauma History	0.02	.041
**Set of nested models 2 (with anxiety and depression)**	NoAge MAS4	.49	69.35	< .001	-242.19		Anxiety	0.29	< .001
							Depression	0.03	.344
	NoAge MAS5	.49	46.22	< .001	-241.95	.481	Anxiety	0.29	< .001
							Depression	0.03	.406
							Childhood trauma	0.00	.488
	NoAge MAS6	.49	34.41	< .001	-241.94	.921	Anxiety	0.29	< .001
							Depression	0.03	.410
							Childhood trauma	0.00	.513
							Impact of trauma history	0.00	.923

Note: NoAgeMAS= Outcome: anxiety sensitivity, without age as a covariate; NoAgeMAS2= predictors: childhood trauma; NoAgeMAS3= predictors: childhood trauma, impact of trauma history; NoAgeMAS4=predictors: anxiety, depression; NoAgeMAS5= predictors: anxiety depression, childhood trauma; NoAgeMAS6= predictors: anxiety depression, childhood trauma, impact of trauma history.

The model comparison showed that in the models without covariates adding impact of trauma history improved the model. In the case of models with covariates, it showed that adding neither childhood trauma or impact of trauma history improved the prediction. In other words, when controlling for anxiety and depression, trauma does not show to explain a significant part of the anxiety sensitivity variance. A summary of the results can be seen in table 6.

Supplementary materials 5: Regression with only age and lifetime trauma as predictors

Model definition	R^2	*F*	*P*	Predictors	β	*p*
Pain catastrophizing ~ age + impact of trauma history	0.07	5.53	0.005	Age	-0.23	0.004
				Impact of trauma history	0.15	0.058

Pain catastrophizing ~ impact of trauma history	0.03	5.97	0.01	Impact of trauma history	0.18	0.01
Anxiety sensitivity ~ age + impact of trauma history	0.08	6.30	0.002	Age	-0.04	0.002
				Impact of trauma history	0.02	0.055
Anxiety sensitivity ~ impact of trauma history	0.03	5.84	0.01	Impact of trauma history	0.02	0.01
